# The Interaction of *Klebsiella pneumoniae* With Lipid Rafts-Associated Cholesterol Increases Macrophage-Mediated Phagocytosis Due to Down Regulation of the Capsule Polysaccharide

**DOI:** 10.3389/fcimb.2019.00255

**Published:** 2019-07-17

**Authors:** Miguel A. Ares, Alejandro Sansabas, Diana Rodríguez-Valverde, Tania Siqueiros-Cendón, Quintín Rascón-Cruz, Roberto Rosales-Reyes, Ma. Dolores Jarillo-Quijada, María D. Alcántar-Curiel, María L. Cedillo, Javier Torres, Jorge A. Girón, Miguel A. De la Cruz

**Affiliations:** ^1^Unidad de Investigación Médica en Enfermedades Infecciosas y Parasitarias, Centro Médico Nacional Siglo XXI, Hospital de Pediatría, Instituto Mexicano del Seguro Social, Mexico City, Mexico; ^2^Facultad de Ciencias Químicas, Universidad Autónoma de Chihuahua, Chihuahua, Mexico; ^3^Unidad de Medicina Experimental, Facultad de Medicina, Universidad Nacional Autónoma de México, Mexico City, Mexico; ^4^Centro de Detección Biomolecular, Benemérita Universidad Autónoma de Puebla, Puebla, Mexico

**Keywords:** *Klebsiella pneumoniae*, cholesterol, capsule, phagocytosis, H-NS, RcsA

## Abstract

*Klebsiella pneumoniae* successfully colonizes host tissues by recognizing and interacting with cholesterol present on membrane-associated lipid rafts. In this study, we evaluated the role of cholesterol in the expression of capsule polysaccharide genes of *K. pneumoniae* and its implication in resistance to phagocytosis. Our data revealed that exogenous cholesterol added to *K. pneumoniae* increases macrophage-mediated phagocytosis. To explain this event, the expression of capsular *galF, wzi*, and *manC* genes was determined in the presence of cholesterol. Down-regulation of these capsular genes occurred leading to increased susceptibility to phagocytosis by macrophages. In contrast, depletion of cholesterol from macrophage membranes led to enhanced expression of *galF, wzi*, and *manC* genes and to capsule production resulting in resistance to macrophage-mediated phagocytosis. Cholesterol-mediated repression of capsular genes was dependent on the RcsA and H-NS global regulators. Finally, cholesterol also down-regulated the expression of genes responsible for LPS core oligosaccharides production and OMPs. Our results suggest that cholesterol plays an important role for the host by reducing the anti-phagocytic properties of the *K. pneumoniae* capsule facilitating bacterial engulfment by macrophages during the bacteria-eukaryotic cell interaction mediated by lipid rafts.

## Introduction

*Klebsiella pneumoniae* is an opportunistic Gram-negative rod-shaped bacterium belonging to the Enterobacteriaceae family that predominantly affects patients with a compromised immune system and is one of the most prevalent causes of nosocomial infections, such as pneumonia, urinary tract infections, meningitis, necrotizing fasciitis, endophthalmitis, pyogenic liver abscess, and sepsis (Podschun and Ullmann, 1998; Alcantar-Curiel and Giron, 2015). In addition, nosocomial isolates of *K. pneumoniae* often display high rates of antimicrobial resistance (Paterson et al., 2004; Ares et al., 2013; Lee et al., 2017; Chong et al., 2018). For a successful infection, *K. pneumoniae* expresses different virulence factors such as capsule, fimbriae, lipolysaccharide (LPS) and outer membrane proteins (OMPs) (Podschun and Ullmann, 1998). *fimA, mrkA*, and *ecpA* genes code for the major pilin subunits of type I, type III, and ECP fimbria, which are involved in the adherence to epithelial cells and formation of biofilms (Struve et al., 2009; Alcantar-Curiel et al., 2013). The transcription of genes responsible for LPS production is mainly driven by two operons, being *wzm* and *rfaD* (also called *hldD*), the two first genes of each transcriptional unit (Li et al., 2014). *wzm* and *rfaD* code for components involved in the O-antigen and core oligosaccharide synthesis, respectively. *K. pneumoniae* expresses three main outer membrane proteins called OmpA, OmpK35, and OmpK36 (Li et al., 2014). The main virulence mechanism of *K. pneumoniae* is resistance to phagocytosis, which is principally due to the capsule polysaccharide at the cell surface that protects the bacterium from opsonization, acts as protective shield against antimicrobial peptides, suppresses the early inflammatory response, and inhibits the maturation of dendritic cells (Cortes et al., 2002; Regueiro et al., 2006; Evrard et al., 2010; Pan et al., 2011; Ko, 2017).

The genes responsible for capsule production are encoded in the *cps* cluster, which is organized in three operons, being *galF, wzi*, and *manC* the first genes of each trancriptional unit (Chou et al., 2004; Chuang et al., 2006; Pan et al., 2011). *galF, wzi*, and *manC* code for UDP-glucose pyrophosphoylase, an outer membrane protein involved in capsule attachment to the cell surface, and mannose-1-phosphate guanylyltransferase, respectively. In terms of regulation, RcsA and H-NS, two chromosome-encoded regulatory proteins, upregulate and repress these capsular genes, respectively (Wehland and Bernhard, 2000; Lin et al., 2011b, 2013; Ares et al., 2016). RcsA forms a regulatory complex with the RcsBCD system, controlling the expression of many genes related with adherence, motility, cell division, biofilm formation and virulence (Majdalani and Gottesman, 2005). H-NS protein binds AT-rich DNA regulatory sequences silencing transcription of housekeeping and virulence genes, acting as a genome sentinel (Dorman, 2007). Moreover, the absence of RcsA or H-NS affects the bacterial adherence to epithelial cells (Ares et al., 2016; Navasa et al., 2019).

Many reports have demonstrated that the host-bacteria contact is mediated by lipid rafts, which are microdomains located on eukaryotic membranes characterized by high concentrations of cholesterol and sphingolipids (Riff et al., 2005; Allen-Vercoe et al., 2006; Lai et al., 2008; Larocca et al., 2010; Matsuda et al., 2010; Lin et al., 2011a; Schiumarini et al., 2017). *K. pneumoniae* requires the presence of lipid rafts located on the macrophages membrane and cholesterol plays an important role in this interaction enhancing phagocytosis (Huang et al., 2013; Cano et al., 2015). This cholesterol-rich microenvironment could affect the expression of virulence factors including capsule. In this work, we determined the effect of cholesterol on the transcription of genes that code for the capsule polysaccharide synthesis in *K. pneumoniae*. The presence of cholesterol negatively affected the expression of the three operons that code for capsule polysaccharide, enhancing macrophage-mediated phagocytosis. The depletion of cholesterol from the macrophage membranes upregulated the expression of *galF, wzi*, and *manC* genes when *K. pneumoniae* was phagocytized, and this effect was RcsA and H-NS-dependent. In addition to capsule polysaccharide, cholesterol repressed the expression of genes that code for both the lipopolysaccharide core and the outer membrane proteins. Our data show that cholesterol exerts a negative effect on the expression of *K. pneumoniae* virulence factors.

## Experimental Procedures

### Bacterial Strains and Growth Conditions

*K. pneumoniae* strains used in this study are listed in Table 1. Bacterial cultures were prepared from overnight Lysogeny Broth (LB) cultures. Bacteria were grown in LB with no supplement, 0.05% tyloxapol (LBT), and 0.05% tyloxapol plus 50 μM cholesterol (LBC). Cultures were grown during 8 h at 37°C shaken at 160 rpm. Ampicillin (200 μg/mL), kanamycin (50 μg/mL), and chloramphenicol (50 μg/mL) were added when required.

**Table 1:** List of bacterial strains and plasmids used.

**Strain or plasmid**	**Genotype or description**	**Reference or source**
***K. pneumoniae*** **strains**		
Kpn WT	Wild-type strain (123/01), serotype K39	Ares et al., 2016
Kpn *rcsA*	Δ*rcsA*::Cm^R^	This study
Kpn *hns*	Δ*hns*::Km^R^	Ares et al., 2016
Kpn *cps*	Δ(*galF-orf2-wzi*)::Km^R^	Ares et al., 2016
**Plasmids**		
pKD119	pINT-ts derivative containing the λ Red recombinase system under an arabinose-inducible promoter, Tc^R^	Datsenko and Wanner, 2000
pKD3	pANTsγ derivative template plasmid containing the chloramphenicol cassette for λ Red recombination, Ap^R^	Datsenko and Wanner, 2000

### Construction of *K. pneumoniae* Δ*rcsA*

The *K. pneumoniae* Δ*rcsA* was obtained using the lambda Red recombinase as previously described (Datsenko and Wanner, 2000). A PCR product was generated using gene-specific primer pairs [CGT GTT GAT TGA GGA TGG GTC ATG TCA ACG ATG ATT ATG GAT TTG TGT AGG CTG GAG CTG CTT CG (rcsA-H1P1) and CGG GAG CGC CGC CAG TTT GTT TCA GCG CAT ATT TAC CTG AAT ACC CAT ATG AAT ATC CTC CTT AG (rcsA-H2P2)], and DNA of the pKD3 plasmid was used as template. This PCR product was electroporated into competent *K. pneumoniae* carrying the lambda-Red recombinase helper plasmid pKD119, whose expression was induced by adding L-(+)-arabinose (Sigma) at a final concentration of 1.0%. The respective mutation was confirmed by PCR using specific primers [CGC AAT CAC GCG CTG CCA CTG GCG GC (rcsA-5′) and GCT GCA CAA ATC CAT AAT CAT CGT TGA C (rcsA-3′)] and sequencing.

### Retrotranscription Quantitative-PCR

Total RNA was extracted from bacteria grown under different culture conditions using the hot phenol method (Jahn et al., 2008). DNA was removed with TURBO DNA-free (Ambion, Inc.) and the quality of RNA was assessed using a NanoDrop (ND-1000; Thermo Scientific) and an Agilent 2100 bioanalyzer with a Picochip (Agilent Technologies). The absence of contaminating DNA was controlled by the lack of amplification products after 35 qPCR cycles using RNA as template. Control reactions with no template and with no reverse transcriptase were run in all experiments. cDNA synthesis and qPCR were performed as previously described (Ares et al., 2016; De la Cruz et al., 2016). Specific primer sequences for genes that code for lipopolysaccharide and outer membrane proteins are shown in Table 2. Primer sequences for capsule (*galF, wzi*, and *manC*) and fimbriae (*mrkA, fimA*, and *ecpA*) genes were previously reported (Ares et al., 2016). 16S rRNA (*rrsH*) was used as a reference gene for normalization and the gene expression was calculated using the 2^−ΔΔ*Ct*^ method (Livak and Schmittgen, 2001). In order to confirm that *rrsH* gene (16S rRNA) was a good endogenous control gen for normalization, absolute quantification was carried out by obtaining a standard curve for such set of primers according to 10-fold dilutions of known amounts of WT *K. pneumoniae* chromosomal DNA (10^5^, 10^6^, 10^7^, 10^8^, 10^9^, and 10^10^ theoretical copies). Crossing threshold (Ct) values were interpolated to standard curve to obtain gene expression (number of gene copies per nanogram of RNA). There was no difference in the expression of *rrsH* gene between each strain or condition tested (Supplementary Figure 1).

**Table 2:** Primers used in this study.

**For qPCR**	
wzm-F	ACG CTG AAC CTG TTT TTC CG
wzm-R	ATA CTC GCT AGC GGA TTG TAG G
rfaD-F	AAG CGC TGA ATG ACA AAG GC
rfaD-R	TGT AGT CAG CGA TGT TCA GGT C
msbA-F	CGG TCT GTT CGT GAT GAT GTT C
msbA-R	TTG CGA AAA CGC TTG GAG AC
ompA-F	ACA CTC AGC TGA GCA ACA TG
ompA-R	AGC TGC TGG TTG TAA GCT TC
ompK35-F	AGC GAC GAT ACC ACC TAT GC
ompK35-R	ACG CGT CCA TGT TGT ATT CC
ompK36-F	CGG TAA AAT TGA CGG TCT GCA C
ompK36-R	GTT GAT CTG GGT TTC GCC TTT C

### Mucoviscosity

The mucoviscosity of *K. pneumoniae* strains was determined as previously described (Lin et al., 2012). Briefly, equal numbers of exponential phase-cultured bacteria were centrifuged at 1,000 g for 5 min. The supernatant was subjected to measurement of the absorbance at 600 nm.

### Glucuronic Acid Analysis

Capsular polysaccharides were extracted and quantified as described (Lin et al., 2009). Bacterial cultures (0.5 mL) were mixed with 100 μl of 1% zwittergent 3–14 in 100 mM citric acid and then the mixtures were incubated at 50°C for 20 min. After centrifugation, 250 μl of supernatants were transferred into new tubes, and 1 mL of absolute ethanol was added to precipitate the capsular polysaccharide. The pellets were dissolved in 200 μL of distilled water, and then 1,200 μL of 12.5 mM borax in concentrated H_2_SO_4_ were added. The mixtures were vigorously vortexed, boiled for 5 min, and then cooled. Twenty microliter of 0.15% 3-hydroxydiphenol in 0.5% NaOH were added to the mixture and the absorbance was measured at 520 nm. The glucuronic acid concentration in each sample was determined from a standard curve of glucuronic acid and expressed in micrograms/10^9^ CFU.

### Cell Adherence Assays

Cultured A549 (ATCC CCL-185) human lung epithelial cell line was used in adherence assays as described (Ares et al., 2016). These monolayer cells (7 × 10^5^) were cultivated in DMEM (Invitrogen) at 37°C under 5% CO_2_ atmosphere in Polystyrene 24-well plates (CellStar). As inoculum, we used *K. pneumoniae* grown in LB, LBT and LBC broths at exponential phase (2 h, OD_600nm_ = 0.8) at 37°C. The cells were infected at a multiplicity of infection (MOI) of 100 for 2 h, washed thrice with PBS to remove unbound bacteria, and subsequently treated with 1 mL of 0.1% TritonX-100 for 15 min. Following lysis, bacteria were quantified by plating out 10-fold dilutions of the bacterial suspensions. Quantifications were performed in triplicate on 3 different days, and the mean results were expressed as adhering CFU/mL.

### Phagocytosis of Bacteria by THP-1 Macrophages

THP-1 (ATCC TIB-202) human monocytes (differentiated to macrophages with 200 nM of phorbol 12-myristate 13-acetate for 24 h) were seeded (6 × 10^5^) into 24-well tissue culture plates. Bacteria were grown in 5 mL of LB, LBT, and LBC to the exponential phase (2 h, OD_600nm_ = 0.8). THP-1 macrophages were infected with a MOI of 100 in a final volume of 1 mL RPMI 1640 tissue culture medium supplemented with 10% heat-inactivated FBS. To synchronize the infection, plates were centrifuged at 200 *g* for 5 min. Plates were incubated at 37°C under a humidified 5% CO_2_ atmosphere. After 2 h, cells were rinsed thrice with PBS and incubated for an additional 1 h with 1 mL of RPMI 1640 containing 10% FBS and gentamicin (100 μg/mL) to eliminate extracellular bacteria. Cells were then rinsed again thrice with 1 mL of PBS and lysed with 1 mL of 0.1% Triton X-100. After homogenization, 10-fold serial dilutions were plated onto LB agar plates to determine total CFU.

When indicated, macrophages were pre-incubated for 1 h with 1 mM methyl-β-cyclodextrin [MβCD (Sigma-Aldrich)], washed twice with PBS to remove cholesterol and infected with *K. pneumoniae*. Treatment with MβCD had no effect on cell and bacterial viability as was described (Cano et al., 2015).

### Biofilm Formation Assay

The biofilm assay was performed as previously described (Saldana et al., 2014). Overnight bacterial cultures were diluted 1:100 with LB, LBT and LBC broths and 200 μL aliquots were transferred to 96-well plates (Nunc, Sigma-Aldrich). After incubation for 24 h at 25°C the medium was discarded and the wells were rinsed thrice with PBS. The bound bacteria were stained with 1% Crystal Violet (Merck). After washing, the adsorbed dye was recovered with ethanol and the color read at an optical density of 595 nm with a spectrophotometer (Multiskan Ascent, Thermo Scientific).

## Results

### Cholesterol Represses Capsule Production

**Figure 1:**
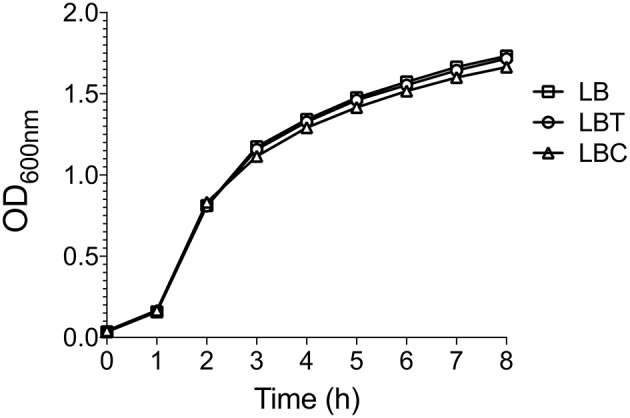
Cholesterol does not affect the *K. pneumoniae* growth. Growth curves of *K. pneumoniae* in LB medium with no supplement (LB), 0.05% tyloxapol (LBT), and 0.05% tyloxapol plus 50 μM cholesterol (LBC). Bacterial cultures were grown at 37°C for 8 h.

**Figure 2:**
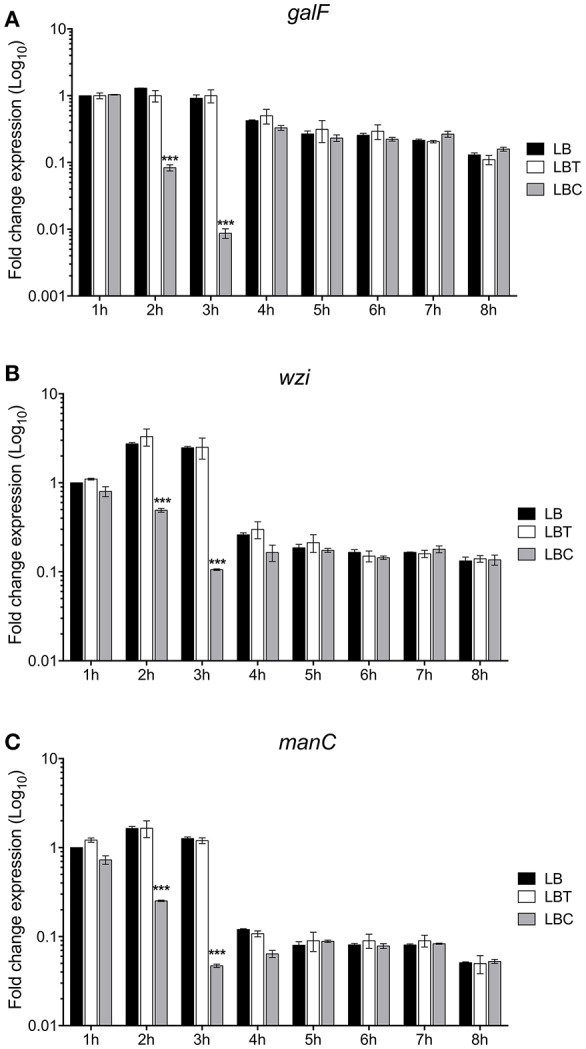
Cholesterol represses the transcription of capsular genes in *K. pneumoniae*. Fold change expression (RT-qPCR) of *galF* **(A)**, *wzi* **(B)**, and *manC* **(C)** genes. *K. pneumoniae* was grown in LB with no supplement (LB), 0.05% tyloxapol (LBT), and 0.05% tyloxapol plus 50 μM cholesterol (LBC). Bacterial cultures were grown at 37°C for 8 h. 16S rRNA was used as a reference gene for normalization. Data represent the mean of three independent experiments performed in triplicates. Statistically significant with respect to the WT bacteria grown in LB medium ***p < 0.001.

To evaluate the effect of cholesterol on the expression of the capsule polysaccharide, *K. pneumoniae* was grown in LB in absence and in presence of cholesterol (tyloxapol was used as surfactant). Neither tyloxapol nor cholesterol affected *K. pneumoniae* growth (Figure 1). To determine gene expression changes in response to 8-h exposure to cholesterol, we analyzed the transcription of capsular *galF, wzi*, and *manC* genes. Interestingly, levels of mRNA of capsule genes were diminished after 2 h of cholesterol contact, reaching higher repression after 3 h (Figure 2). At this time point, *galF, wzi*, and *manC* were repressed 105-, 23-, and 27-fold, respectively, when *K. pneumoniae* was grown with cholesterol (Figure 2). No differences were found after 4 h of cholesterol contact.

**Figure 3:**
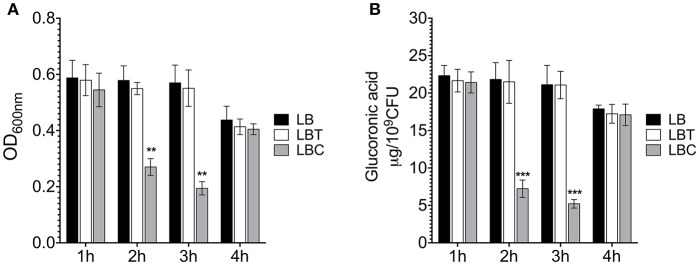
Cholesterol affects the production of capsular polysaccharide in *K. pneumoniae*. **(A)** Mucoviscosity of *K. pneumoniae* grown in LB with no supplement (LB), 0.05% tyloxapol (LBT), and 0.05% tyloxapol plus 50 μM cholesterol (LBC). The mucoviscosity was determined by low speed centrifugation and is expressed as OD_600nm_ of the supernatant. **(B)** Capsule quantification by determination of the glucuronic acid concentration from capsular polysaccharides. Statistically significant with respect to the WT bacteria grown in ** *p* < 0.01 between LB medium and ***p < 0.001.

To corroborate the effect of cholesterol on the production of the capsule polysaccharide in *K. pneumoniae*, we compared levels of mucoviscosity and the amounts of capsular glucuronic acid among the strains. Similar to transcription (Figure 2), cholesterol effect on translation was observed after 2 h of treatment with this lipid, observing a reduction of capsule production (3-fold) (Figure 3B). Cholesterol diminished mucoviscosity and capsular glucuronic acid 3- and 4-fold, respectively, after 3 h of growth (Figure 3). These data show that the transcription/production of the capsule polysaccharide in *K. pneumoniae* is negatively affected by cholesterol.

### Cholesterol From Macrophages Lipids Rafts Represses the *K. pneumoniae* Capsule Polysaccharide

**Figure 4:**
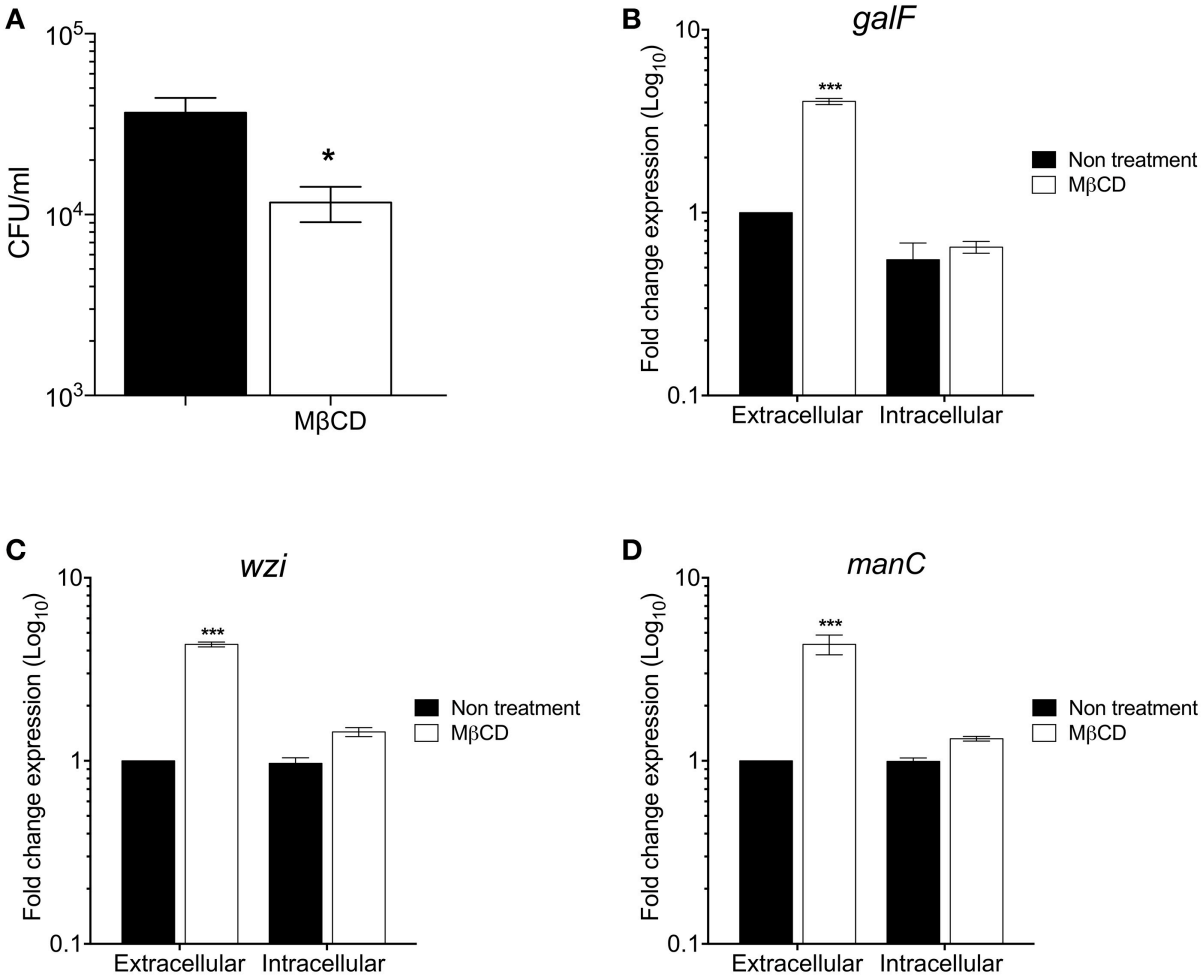
The depletion of cholesterol from macrophages lipids rafts increases the *K. pneumoniae* capsule polysaccharide. **(A)** Comparison of phagocytic uptake of *K. pneumoniae* by THP-1 macrophages treated without/with MβCD (methyl-β-cyclodextrin) to deplete cholesterol. Fold change expression (RT-qPCR) of *galF* **(B)**, *wzi* **(C)**, and *manC* **(D)** genes of *K. pneumoniae* (extra and intracellular) during the phagocytosis by THP-1 macrophages treated without/with MβCD. Data represent the mean of at least three independent experiments performed in triplicates (mean ± SD). Statistically significant with respect to the WT bacteria grown in LB medium * *p* < 0.05; *** *p* < 0.001.

To investigate the role of cholesterol from lipids rafts on the macrophage-mediated phagocytosis, methyl-β-cyclodextrin (MβCD) was employed to deplete cholesterol from macrophage-cell membranes (Figure 4A). Cholesterol depletion diminished phagocytosis of *K. pneumoniae* as previously reported (Huang et al., 2013; Cano et al., 2015). In addition, we analyzed the effect of cholesterol depletion from lipids rafts on the transcription of *galF, wzi*, and *manC* genes in both extra and intracellular bacteria during phagocytosis (Figures 4B–D). Transcription of *galF, wzi*, and *manC* genes increased 4-fold in extracellular *K. pneumoniae* when cholesterol was depleted from macrophage membranes (Figures 4B–D). However, expression of capsule genes in intracellular *K. pneumoniae* was not affected in macrophages treated with MβCD. These data indicate that cholesterol present in lipids rafts represses capsule expression enhancing the phagocytosis.

### Role of Cholesterol on Phagocytosis and Adherence

**Figure 5:**
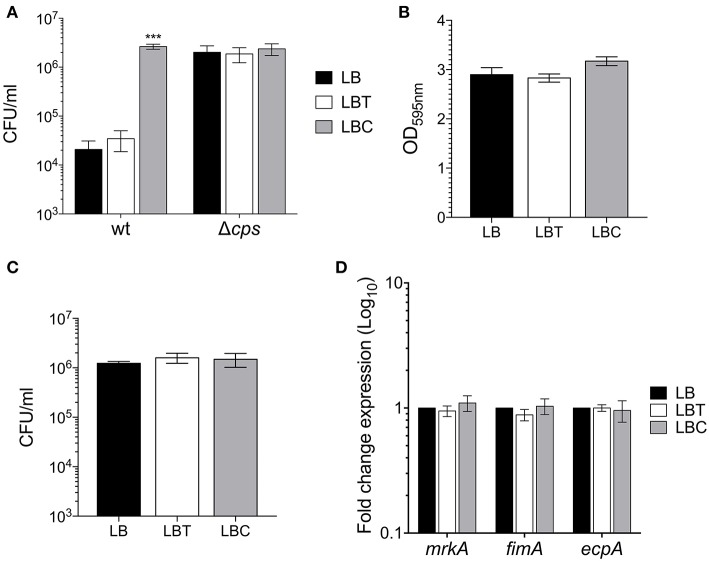
Cholesterol enhances macrophage-mediated phagocytosis of *K. pneumoniae*. **(A)** Comparison of phagocytic uptake of *K. pneumoniae* grown under the indicated different conditions by THP-1 macrophages. A Δ*cps* mutant was evaluated as control of phagocytosis. **(B)** Quantification of biofilm formation by measuring Crystal Violet uptake of *K. pneumoniae* in LB, LBT and LBC broths. **(C)** Adherence levels of *K. pneumoniae* grown in LB, LBT and LBC broths to A549 cells. **(D)** Fold change expression (RT-qPCR) of fimbrial genes. 16S rRNA was used as a reference gene for normalization. Data represent the mean of at least three independent experiments performed in triplicates (mean ± SD). Statistically significant with respect to the WT bacteria grown in LB medium ***p < 0.001.

A hallmark of the pathogenesis of *K. pneumoniae* is its resistance to macrophage-mediated phagocytosis, due to the capsule polysaccharide. Given that cholesterol reduced capsule expression (Figures 3, 4), we evaluated whether exposure of bacterium to cholesterol would have an effect on phagocytosis. The bacteria grown in presence of cholesterol were readily phagocytized (126-fold) by THP-1 macrophages as compared to bacteria without cholesterol (Figure 5A), corroborating that these bacteria produced very little capsule. As a control of phagocytosis, a Δ*cps* mutant [a mutant that does not form a capsule since it carries a deletion Δ(*galF-orf2-wzi*), (Ares et al., 2016)] was included. The lack of capsule dramatically increased macrophage-mediated phagocytosis as previously described (Ares et al., 2016) and these levels were as high as in cholesterol-pretreated bacteria. These observations show that the effect negative of cholesterol on the macrophage-mediated phagocytosis of *K. pneumoniae* was capsule-dependent (Figure 5A).

In addition, we analyzed the effect of cholesterol on the adherence of *K. pneumoniae* to both abiotic (biofilm formation) and biotic surfaces (adherence to epithelial cells). Premixing of the bacteria with cholesterol did not alter biofilm formation nor adherence to A549 epithelial cells (Figures 5B,C). Moreover, the transcription of fimbrial genes (*fimA, mrkA*, and *ecpA*), which are required for either biofilm formation or adherence to epithelial cells (Langstraat et al., 2001; Alcantar-Curiel et al., 2013; Ares et al., 2016; Hsieh et al., 2016), was not affected in presence of this lipid (Figure 5D). In summary, the data strongly suggest that cholesterol is not involved in adherence or adherence factors of *K. pneumoniae*.

### The Negative Effect of Cholesterol on *cps* Genes Is RcsA- and H-NS-Dependent

**Figure 6:**
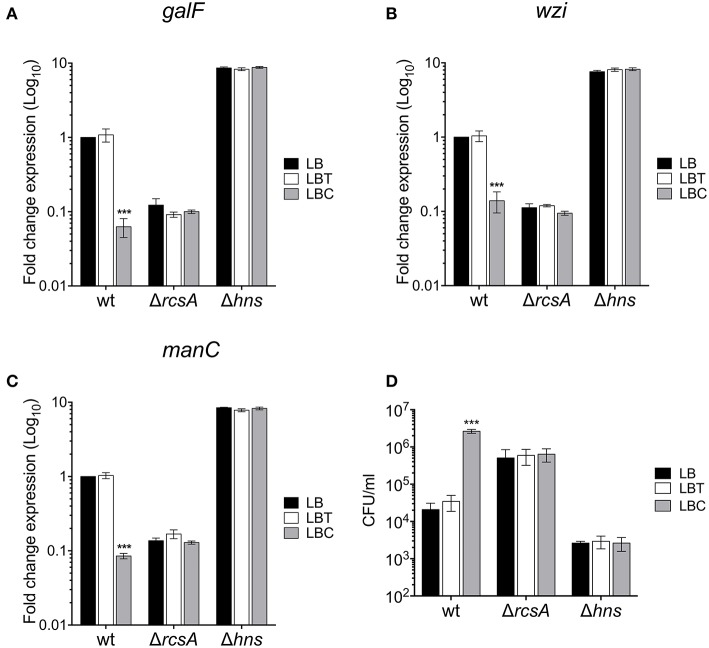
RcsA and H-NS are involved in the cholesterol-mediated capsule repression. Fold change expression (RT-qPCR) of *galF* **(A)**, *wzi* **(B)**, and *manC* **(C)** genes in the presence of cholesterol [LB with no supplement (LB), 0.05% tyloxapol (LBT), and 0.05% tyloxapol plus 50 μM cholesterol (LBC)] in different backgrounds: wild-type, Δ*rcsA* and Δ*hns*. 16S rRNA was used as a reference gene for normalization. **(D)** Effect of cholesterol on phagocytic uptake of *K. pneumoniae* wild-type, Δ*rcsA*, and Δ*hns* by THP-1 macrophages. Data represent the mean of at least three independent experiments performed in triplicates (mean ± SD). Statistically significant with respect to the WT bacteria grown in LB medium ***p < 0.001.

RcsA and H-NS act as positive and negative regulators of the *cps* genes, respectively (Wehland and Bernhard, 2000; Lin et al., 2011b, 2013; Ares et al., 2016). To determine if these regulatory proteins were involved in cholesterol-mediated repression of capsule genes, the expression of *galF, wzi*, and *manC* was compared in the wild-type, Δ*rcsA* and Δ*hns* mutants, in response to addition of cholesterol to LB (LBC). Indeed, the expression of all three capsule genes was positive and negatively regulated by RcsA and H-NS, respectively (Figures 6A–C). Interestingly, cholesterol did not affect the transcription of capsule genes in the absence of these regulatory proteins (Figures 6A–C). We also analyzed the role of RcsA and H-NS in resistance to phagocytosis. Similar to the Δ*cps* mutant (Figure 2A), the absence of RcsA increased (25-fold) levels of phagocytosis (Figure 6D). In contrast to Δ*rcsA*, a Δ*hns* mutant showed high resistance to phagocytosis as previously reported [Figure 6D; (Ares et al., 2016)]. Compared to wild-type strain, the number of Δ*rcsA* and Δ*hns* mutants inside macrophages was not increased when they were grown in the presence of cholesterol (Figure 3D). Our data show that cholesterol-mediated signaling on the capsular polysaccharide involves RcsA and H-NS.

### Role of Cholesterol on Other *K. pneumoniae* Virulence Factors

**Figure 7:**
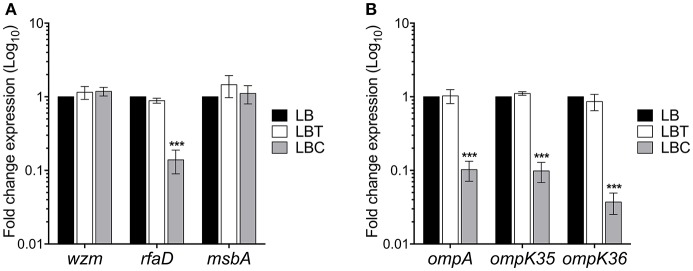
Cholesterol affects other virulence genes in *K. pneumoniae*. Fold change expression (RT-qPCR) of genes responsible for LPS production **(A)** and outer membrane proteins **(B)**. *K. pneumoniae* was grown in LB with no supplement (LB), 0.05% tyloxapol (LBT), and 0.05% tyloxapol plus 50 μM cholesterol (LBC) at 37°C for 3 h. 16S rRNA was used as a reference gene for normalization. Data represent the mean of three independent experiments performed in triplicates. Statistically significant with respect to the WT bacteria grown in LB medium ***p < 0.001.

We then analyzed the role of cholesterol on other surface components such as lipopolysaccharide (LPS) and outer membrane proteins (OMPs). LPS transcription is mainly driven by two operons, *wzm* and *rfaD* (Li et al., 2014). Whilst *wzm* was not affected, *rfaD* transcription was repressed 7-fold in the presence of cholesterol (Figure 7A). In addition, the analysis of the expression of *msbA*, which codes for an ABC transporter involved in the lipid A synthesis, was not affected by cholesterol. The main OMPs of *K. pneumoniae* are OmpA, OmpK35, and OmpK36, which are encoded in monocistronic units (Li et al., 2014). Cholesterol negatively affected the expression of *ompA* (10-fold), *ompK35* (10-fold), and *ompK36* (27-fold) (Figure 7B). Our data show that in addition to capsule polysaccharide, cholesterol also affects the transcription of both LPS core oligosaccharide and the OMPs.

## Discussion

The capsule polysaccharide is the main virulence factor of *K. pneumoniae*. The overproduction of this complex structure is associated with the hypervirulence of this group of bacteria (Lee et al., 2017; Dorman et al., 2018). Eukaryotic cell-bacteria contact is crucial not only in the virulence of *K. pneumoniae*, but in general in bacterial pathogenesis. Lipid rafts are microdomains located on the eukaryotic membranes with high concentrations of sphingolipids and cholesterol, and function in a different process such as signal transduction, receptors for microbial recognition, trafficking and providing stability to cell membranes (Zajchowski and Robbins, 2002; Helms and Zurzolo, 2004; Kwiatkowska, 2010; Lingwood and Simons, 2010; Reeves et al., 2012). Many reports have shown that macrophage-mediated phagocytosis of *K. pneumoniae* is an event dependent on the lipid rafts since cholesterol depletion from eukaryotic membranes impaired *K. pneumoniae* engulfment by macrophages (Huang et al., 2013; Cano et al., 2015). Here, our data indicate that cholesterol also represses both expression and production of the *K. pneumoniae* capsule polysaccharide. The *K. pneumoniae*'s transcriptional response to cholesterol occurred 2 h after exposition to this lipid and it was maintained until 3 h, suggesting temporal expression of *cps* genes in the presence of cholesterol. At 4 h, cholesterol could be metabolized or modified (i.e., esterification) by bacterial enzymes, eliminating the negative effect of this lipid on the capsule polysaccharide expression/production. The effect of cholesterol on *cps* expression was not due to the disruption of the capsule, as shown by the lack of a similar effect of the tyloxapol surfactant.

The depletion of cholesterol from macrophage cell membranes caused an increase in transcription of *cps* promoters, stimulating the production of the capsule polysaccharide and consequently diminishing phagocytosis. We also found that production of the capsule was not enhanced under intracellular conditions. These data highlight the need of this virulence factor against phagocytosis and confirms the role of lipid rafts cholesterol in regulation of capsule production. Thus, it appears that cholesterol on lipid rafts is an important self-defense mechanism for host cells, specifically macrophages, against organisms that possess an anti-phagocytic capsule. In addition to lipids rafts, another source of cholesterol in the context of systemic infection caused by *K. pneumoniae* could be the cholesterol-transporters lipoproteins circulating in blood such as VLDL, LDL, and HDL. The relevance of these molecules in the regulation of *K. pneumoniae* capsule remains unknown. In terms of the binding of *K. pneumoniae* to biotic and abiotic surfaces, the presence of cholesterol did not repress biofilm formation or adherence to epithelial cells, supporting the notion that: (i) cholesterol does not affect the expression of fimbrial genes, and (ii) capsule polysaccharide is not required for adherence to both biotic and abiotic surfaces, as previously reported (Ares et al., 2016).

RcsA and H-NS are two regulatory proteins acting as activators and repressors of the capsule transcription, respectively (Wehland and Bernhard, 2000; Lin et al., 2011b, 2013; Ares et al., 2016). The cholesterol-mediated signaling sensed by *K. pneumoniae* to control the *cps* genes was RcsA- and H-NS-dependent. Furthermore, the macrophage-mediated phagocytosis of both Δ*rcsA* and Δ*hns* mutants was not altered when these bacteria were in contact with cholesterol during growth. A recent paper showed the complexity of regulatory networks involved in the *K. pneumoniae* capsule transcription, describing the presence of transcription regulators such as ArgR, MprA, and SlyA (Dorman et al., 2018). Future directions of this work will evaluate the role of these regulators as proteins that sense and/or integrate cholesterol-mediated signaling that repress *cps* genes in *K. pneumoniae*.

Interestingly, cholesterol also affected the expression of both LPS core oligosaccharide and OMPs. Although both virulence factors have been described as players in the macrophage-mediated phagocytosis of *K. pneumoniae*, previous observations and our results corroborate the main role of capsule polysaccharide in *K. pneumoniae* pathogenesis (Insua et al., 2013; Cano et al., 2015).

In summary, our results show the relevance of cholesterol as a signaling molecule that negatively affects the transcription/production of the capsule polysaccharide, which is the main virulence factor of *K. pneumoniae*, participating in the resistance of this bacterium to phagocytosis.

## Data Availability

All datasets generated for this study are included in the manuscript and/or the Supplementary Files.

## Author Contributions

MA and MD conceived and designed the experiments. MA, AS, DR-V, RR-R, MJ-Q, and MC performed the experiments. MA, TS-C, QR-C, MA-C, JT, JG, and MD analyzed the data. MA, JG, and MD wrote the paper.

### Conflict of Interest Statement

The authors declare that the research was conducted in the absence of any commercial or financial relationships that could be construed as a potential conflict of interest.

## Supplementary Material

Supplementary Figure 1 Expression of reference gene (16S rRNA) under different growth conditions. Panels show the expression of reference gene under different growth conditions, such as expression kinetic in LB, LBT, and LBC **(A)**, gene expression of *K. pneumoniae* (extra and intracellular) during the phagocytosis by THP-1 macrophages treated without/with MβCD **(B)**, and gene expression in different backgrounds (wild-type, Δ*rcsA*, and Δ*hns*) in LB, LBT, and LBC **(C)**. Quantification of expression is showed as copies of *rrsH*/μg RNA.
