# Supplementary figures and images for: The Interaction of Klebsiella pneumoniae With Lipid Rafts-Associated Cholesterol Increases Macrophage-Mediated Phagocytosis Due to Down Regulation of the Capsule Polysaccharide
Source: Front Cell Infect Microbiol. 2019 Jul 17;9:255. doi: 10.3389/fcimb.2019.00255 (PMC6650577; doi:10.3389/fcimb.2019.00255)

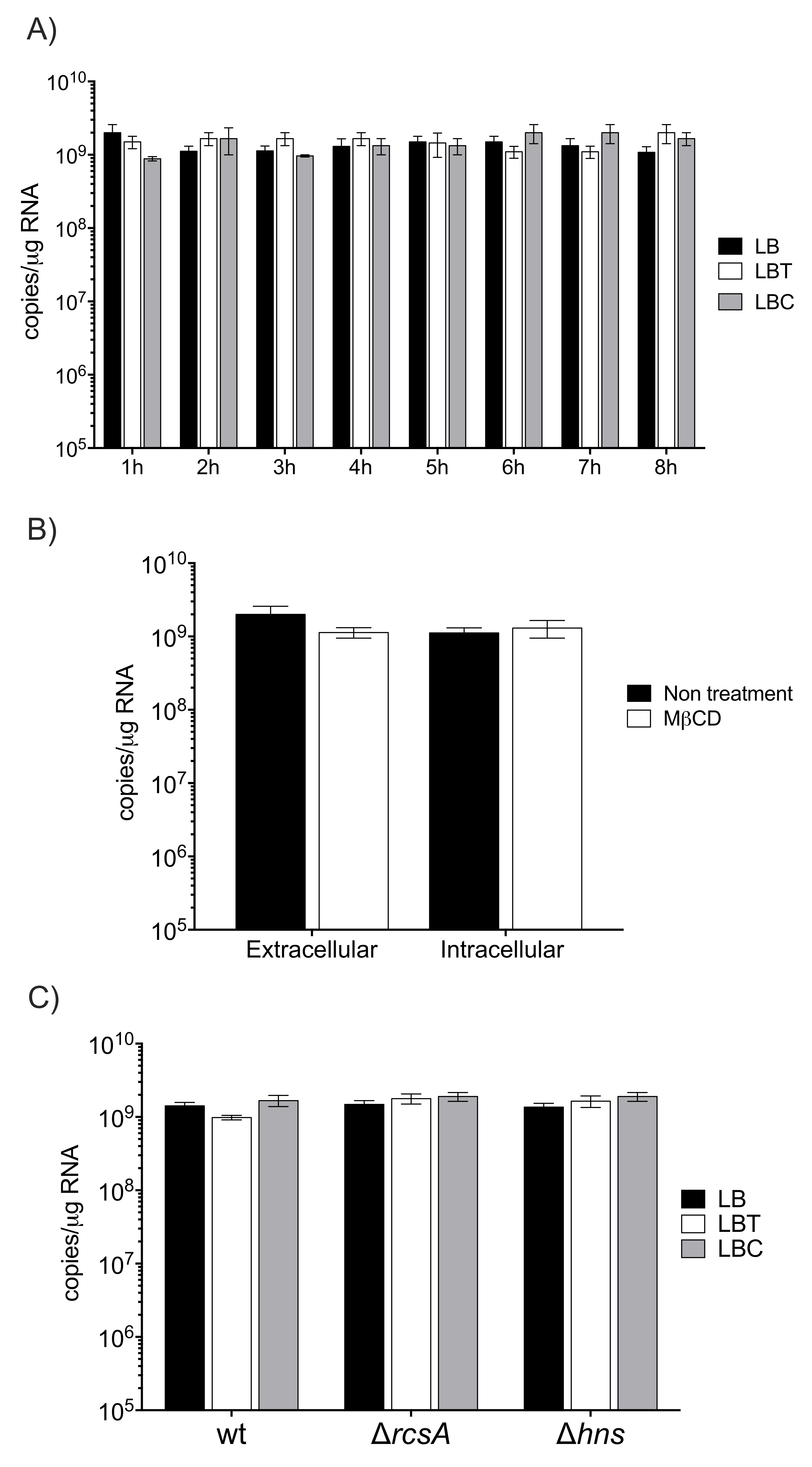

Supplement: Supplementary Figure 1 — Expression of reference gene (16S rRNA) under different growth conditions. Panels show the expression of reference gene under different growth conditions, such as expression kinetic in LB, LBT, and LBC (A), gene expression of K. pneumoniae (extra and intracellular) during the phagocytosis by THP-1 macrophages treated without/with MβCD (B), and gene expression in different backgrounds (wild-type, ΔrcsA, and Δhns) in LB, LBT, and LBC (C). Quantification of expression is showed as copies of rrsH/μg RNA. [file Image_1.TIFF]
